# 

**Published:** 1904-03-05

**Authors:** 


### CONTENTS.

**Ambulances for London** - - - - - - - - 399

**Lady Health Inspectors in Kensington** - - - 400

**Annotations** - - - - - - - - 401

**Notes and News** - - - - - - - - - 402

**Hospital Clinics and Medical Progress—**

The Sanatorium Treatment of Consumption - - - - 403

Treatment of Hepatic Abscess - - - - - - - 405

The History of Patients after Gastric Operations - - - 405

Lymphatic Glands in the Cheek - - - - - - 405

PROGRESS IN FEVERS - - - - - - - - - - 406

PROGRESS IN BACTERIOLOGY - - - - - - - - 408

PROGRESS IN DISEASES OF THE BLOOD - - - - - - - 409

**Hospital Administration—**

The New Science Buildings at Cambridge - - - - - 410

Will London Awake? - - - - - - - - - - 412

Ambulances for London - - - - - - - - - 413

Hospital Meetings - - - - - - - - - - 415

*NURSING SECTION.*

**Notes on News from the Nursing World—**
Princess Christian's Nurses—Threatened Withdrawal of Nurses in East London—The Matron's Signature on Certificates—Making it Hard for Junior Nurses—The Nurses of the Evelina Hospital—The Proposed Amendment of the Midwives Act—Nurses advised to Ask for Credit—Retrogression in Workhouse Nursing—Auckland Working Men's Nursing Association—Women Nurses in Wards for Insane Men—A Dubious Experiment at Southport—Dismissals at Beverley Workhouse Infirmary—Swansea Hospital and Private Nursing—The other Registration Bill - - 305-308
**The Nursing Outlook** - - - - - - 309
**Lectures on Ophthalmic Nursing** - - - - - 310
**Hospital and Private Nursing in Madeira** - - 311
**Maternity Work in South Africa: Nursing in a Dutch Family** - - - - - - - - 312
**Everybody's Opinion** - - - - - - - - 313
**Presentations** - - - - - - - - - 314
**Travel Notes and Queries** - - - - - - - 314
**Appointments** - - - - - - - - - 314
**The Nurses' Bookshelf** - - - - - - - - 314
**Where to Go** - - - - - - - - - 314
**Echoes from the Outside World** - - - - - - 315
**Notes and Queries** - - - - - - - - 316
**For Reading to the Sick** - - - - - - - 316

